# Diapausing Cavity-Nesting Bees (*Osmia, Megachile*) Resist Winter Desiccation Stress

**DOI:** 10.3390/insects16090946

**Published:** 2025-09-10

**Authors:** James H. Cane

**Affiliations:** 1 USDA-ARS (Agricultural Research Service) Pollinating Insect Research Unit, Utah State University, Logan, UT 84322, USA; jim.cane2@gmail.com; 2 WildBeecology Consulting, Logan, UT 84341, USA

**Keywords:** *Osmia lignaria*, *Megachile rotundata*, humidity, diapause, apiformes, climate

## Abstract

All solitary and primitively eusocial bees of the temperate zones survive winter in a dormant diapausing state. Weight loss of *Osmia* and *Megachile* bees has been solely attributed to metabolism of stored triglycerides, as evidenced by loss of fat body. I hypothesized that uptake or loss of water vapor may represent another contributor to wintering weight change. Using a range of atmospheric humidities (0–88%), it is shown that wintering cocooned *O. californica* (adults) and *M. rotundata* (prepupae) gained weight in humid atmospheres and lost weight in drier air, the inflection point being between 35 and 47% humidity. Most of this weight change occurred in early winter. No humidity treatment enhanced mortality. Winter storage humidity is an available management variable for these bees when kept for commercial pollination.

## Simple Summary

All bees that produce annual nests in the temperate latitudes pass the cold winter season as either post-feeding larvae or, less commonly, developed adults. Among the non-social species are two genera of cavity-nesters that are managed for crop pollination (e.g., the alfalfa leaf-cutting bee and several mason bees). Their weight loss over winter has been attributed to fat metabolism. In this study, we also show that the wintering stages gain or lose weight as a function of atmospheric humidity. Wintering bees handily survived even extreme humidities (0 and 88%), giving bee managers latitude for coldroom storage conditions for their bee stocks.

## 1. Introduction

Solitary (non-social) bees of the temperate zones pass the colder winter months as either a pre-emergent adult or a post-feeding larva (prepupa) within their natal brood cell. During this time, they do not defecate, thus retaining both accumulated metabolic waste and metabolic water. During their winter dormant stages, they do not feed either, so winter water loss or gain must be respiratory or possibly trans-cuticular. For the few species that have been investigated, their metabolic rates are suppressed in early winter by physiological diapause and thereafter by ambient cold. Most solitary bee species nest underground, where winter soil temperatures are benign and atmospheric humidities should be saturated [1]. Other species nest above-ground, occupying tunnels in deadwood or pithy twigs, or even within free-standing nests. Many live in arid regions. Wintering humidities around brood cells in these above-ground contexts can be expected to be less than saturated, but actual values have not been measured.

Several cavity-nesting bee species of the family Megachilidae are managed in man-made nesting substrates to pollinate temperate-zone crops. The alfalfa leaf-cutting bee *Megachile rotundata* is the primary commercial pollinator of alfalfa (lucerne) and several other seed crops (e.g., canola) [2], whereas the three to four managed species of subgenus *Osmia (Osmia)* can be used to pollinate rosaceous tree fruits (e.g., almond, apple, cherry, pear) [3]. Dormant populations are typically wintered in refrigerators or coolers set to 3–4 °C [4], although outdoor storage is favorable too [5]. Saturated humidities are avoided for indoor winter storage of these bees [3]. Conversely, when *M. rotundata* bees were being incubated in early summer for adult emergence, lower humidities (<40%) were lethal [6]. Otherwise, the wintering humidities experienced by diapausing solitary bees and their consequences are not reported from the field and are generally not considered during experiments or management.

Winter weight loss and mortality are typically attributed solely to the gradual depletion of fat reserves experienced by all wintering individuals. In this study, the contribution of desiccation stress to bees’ overwintering performance and weight loss is experimentally evaluated. I hypothesized that ambient humidity would contribute to weight changes in dormant bees, which should vary among their deadwood nesting substrates. Winter weight gains would clearly implicate water uptake, given the known weight losses attributed to winter metabolism of fat body reserves. I expected that few bees would survive extreme aridity.

## 2. Materials and Methods

### 2.1. Life Cycle of Osmia and Megachile

Like most bees, both genera consist of non-social species whose larvae are individually fed a mass provision of pollen moistened with nectar. Many *Osmia* and *Megachile*, including *O. californica* and *M. rotundata*, nest in above-ground cavities, typically occupying the tunnels left in deadwood by wood-boring beetles. Like most other solitary bees, immature *M. rotundata* pass the winter as a post-feeding larva (prepupa). In contrast, species of *Osmia* pupate and eclose as an adult within their cocoon, where they remain for the winter months. Like other megachilids, their larvae spin tough cocoons within which the individual passes the winter.

### 2.2. Sources and Preparation of Bees

Nests of *O. californica* were obtained from dry cut reeds placed out in the foothills of the Bear River Range in northern Utah and adjacent Idaho, USA. Their floral host is *Balsamorhiza sagittata* (Asteraceae). Nests of *M. rotundata* were started from commercial Canadian loose-cell stock produced on alfalfa (*Medicago sativa*: Fabaceae). These populations were then used to locally rear another generation the following year. Those nests were recovered from the field in late summer. They were held at shaded ambient conditions in Logan into the autumn. Cocoons were removed from their nests and manually cleaned of loose detritus, frass, nest cell partitions, and most of the leaf pack (*M. rotundata*) to eliminate extraneous materials that could adsorb or lose moisture. Cocoon contents were then revealed using X-ray imaging to confirm development and to identify healthy progeny. Visibly damaged cocoons were discarded. Cocooned bees were then held at 4 °C and 26% relative humidity in the weeks prior to beginning experimental humidity treatments on December 6.

### 2.3. Experimental Manipulation of Wintering Humidities

Sixteen cocoons of each species were placed in each uncovered 48-well ELISA plate, and there were six plates in total (total of 96 cocoons per species). Each ELISA plate with its cocoons was placed in a separate 1.3 L plastic tub with a rubberized lid seal (“Lock-its”^TM^, Rubbermaid, Homerville, NC, USA). To evaluate the role the cocoon plays as a barrier to water loss, a small hole was cut in the tip of some cocoons using a surgical scalpel. For each of the 16 wintering bees, four cocoons per species and treatment were thus cut (only females in the case of *O. californica*) to directly expose wintering individuals to the range of treatment humidities.

Fixed humidities were maintained over an open pan in each container that held either an oven-dried desiccant or a saturated salt solution (Winston and Bates, 1960). Expected relative humidities (and their desiccant or their saturated salt solution) were **0%** (Drierite^TM^ desiccant), **5%** (NaOH), **35%** (MgCl_2_), **47%** (K_2_CO_3_), **75%** (NaCl), and **88%** (KCl). After five days equilibrating at 5 °C, actual relative humidity in each tub was measured at 5 °C using a custom digital humidity meter fitted with a HMP35A sensor (HMP, Helsinki, Finland). Its design accurately measures a wide range of relative humidities in cold air (Apogee Instruments, Logan, UT, USA). Measured humidities in the containers were 1.5, 5.5, 35, 55, 75, and 88%, with only the K_2_CO_3_ deviating slightly from expectation.

Chilled bees in their natal cocoons were initially weighed (Mettler AC 100 balance) on **6 December** and then set in the controlled humidity tubs. Thereafter, cocoons with bees were individually weighed every six weeks during the winter. To maintain chill and minimize atmospheric moisture exchange while weighing cocoons, the well plate was set atop an ice pack and kept covered. In April, at the end of the wintering period, bees were warmed to 20 °C for 3 d, then incubated at 30 °C and 30% RH to await adult emergence. Bee survival was scored at emergence.

### 2.4. Statistical Analyses

Weight changes of wintering bees in their cocoons were compared by species across the spectrum of humidities using generalized linear mixed models (Proc Mixed [7]). Final April weights were compared using an analysis of covariance with initial weight as the covariate. For wintering female *O. californica*, weights of females at 0% humidity varied much more than those at other humidities. Heterogeneous variances were partitioned by computing separate variances for RH = 0 and for the group of other treatment humidities. Initial water contents of bee species and sex were compared using a Mann–Whitney U-test. Overwintering weight changes of bees in intact versus nicked cocoons were compared across humidity treatments by paired *t*-test. The percent changes in overwintering live weights were regressed on storage humidity by species (SigmaPlot 12.5, Grafiti, Palo Alto, CA, USA.). Winter mortalities by humidity treatment were scored during adult emergence. Initial weights of bees that had died or survived were compared using a Mann–Whitney Rank Sum test.

### 2.5. Auxiliary Treatments

Moisture gain or loss was measured for two groups of ten 1-year-old empty cocoons of *O. californica*. These were placed in 35% humidity to equilibrate for 3 d. After weighing, they were then placed at either 0% or 88% humidity for a week, then reweighed. Moisture gains or losses of the empty cocoons were calculated from the weight changes.

The bodily water contents of both bee species were measured at the start of their dormancy season. Ten live adult *O. californica* (five of each sex) and ten *M. rotundata* prepupae were removed from their cocoons in mid-October, killed, weighed, dried for 48 h at 80 °C, and reweighed to calculate the fraction of body weight that was water. Median species differences in body water content of prewintering *O. californica* and *M. rotundata* were compared using a Mann–Whitney Rank Sum test.

### 2.6. Wintering Humidities in Deadwood Cavities

Humidities were measured in tunnel cavities of dead sound trees during the bees’ winter diapause season. The tree species chosen (*Populus tremuloides, Pseudotsuga menziesii, Pinus contorta*) all host wood-boring beetles whose emergence holes are used by nesting *Osmia* bees. The appearance and condition of the nine standing and two downed dead tree trunks resembled those used by the authors for annual trap-nesting of *Osmia* species.

Accurately measuring humidity in small spaces like bee brood cells and nest tunnels is not possible, given the large size of sensitive instrumental humidity sensors. We therefore drilled 1.2 cm diameter holes radially to a typical nesting depth of 8 cm into sound wood of logs ranging in circumferences from 75 to 176 cm. This hole diameter snugly fit a humidity sensor known for its accuracy at high humidities (HMP50-L, Campbell Scientific, Logan, UT, USA). The fresh drill hole was first corked for 30 mins so its trapped air would equilibrate (as periodically checked by sensor readings). The sensor was then slipped into the drill hole and its lead wire sealed in place with a clay disk. Once the sensor output had stabilized, the relative humidity of the cavity was recorded. To estimate wood water content, wood chips from drilling were collected, sealed up, weighed fresh, dried at 70 °C for 48 h, and reweighed.

## 3. Results

At the start of winter dormancy, water constituted about half of an immature bee’s body weight (Table 1). Both sexes of *O. californica* had equivalent body water contents (Table 1), which was proportionally more than that of *M. rotundata* (Mann–Whitney U-test T = 57, *p* < 0.001).

**Figure 1:**
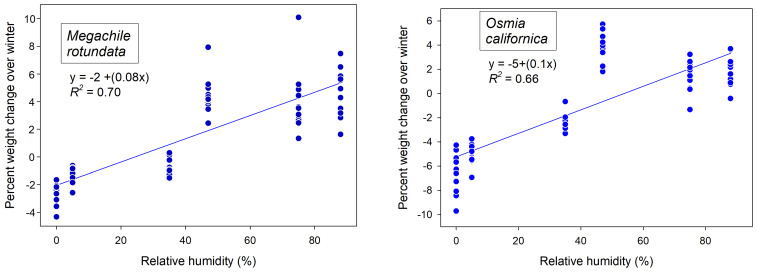
Overall percent change in winter live weights of 72 diapausing *M. rotundata* prepupae (**a**) and 72 adult cocooned *O. californica* (**b**) held at different fixed relative humidities.

**Figure 2:**
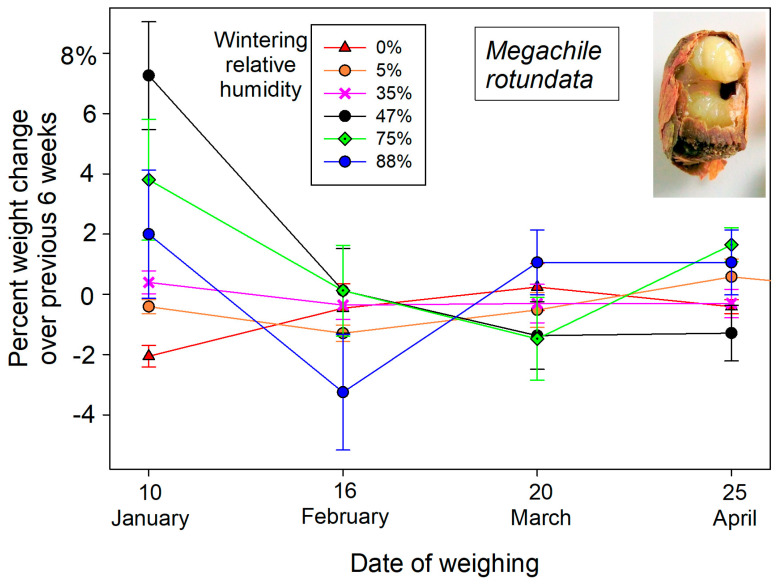
Percent weight change every six weeks of dormant *M. rotundata* prepupae wintering at a range of relative humidities. Means and standard deviations are plotted. *Inset*. Cocoon cut open to reveal the wintering prepupa within.

**Figure 3:**
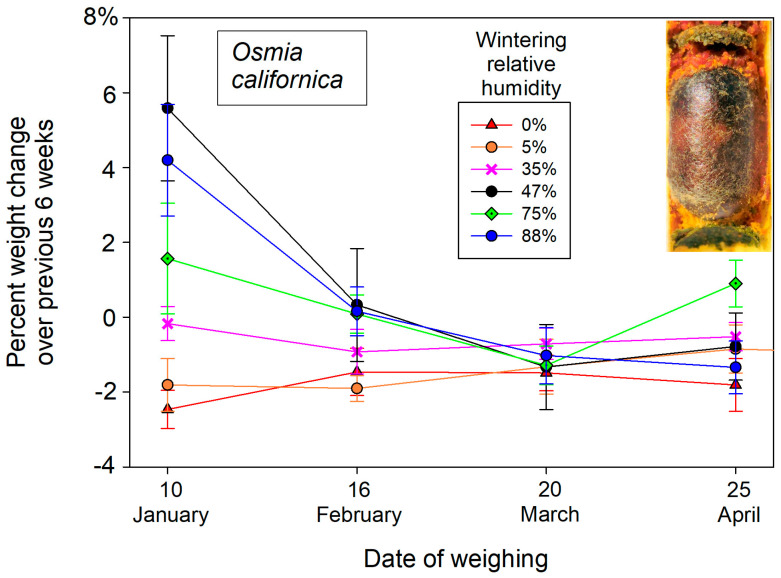
Percent weight change every six weeks of dormant *O. californica* adults wintering at a range of relative humidities. Means and standard deviations are plotted. *Inset.* Cocoon containing an adult *O. californica* and brood cell partitions made from masticated leaf.

These initial weights of dormant bees changed over the winter months in response to atmospheric humidity exposure. Overwintering cocooned bees lost weight in dry atmospheres and gained weight in humid cold storage (Figure 1). The post-wintering weights of *M. rotundata* prepupae responded to wintering humidity (F_5,84_ = 4.17, *p* = 0.002) after accounting for individual starting weights in the mixed model ANCOVA. Wintering humidity likewise contributed to weight changes of female *O. californica* (F_5,21.6_ = 5.78. *p* = 0.0015) but not males (F_5,24_ = 2.11, *p* = 0.098) in the mixed model ANCOVA. Most weight gain or loss at different wintering humidities occurred during the first six weeks (early December into late January) (Figure 2 and Figure 3). The percent changes in overwintering live weights as functions of storage humidity could be linearly represented for the pooled sexes of both *O. californica* (*R*^2^ = 0.66, *p* < 0.0001) and *M. rotundata* (*R*^2^ = 0.69, *p* < 0.0001) (Figure 1). For both species, the inflection point between weight loss and weight gain over the winter was between the 35% and 47% relative humidity treatments (Figure 1).

Cocoons did not inhibit water vapor exchange for the bee within. Thus, the average wet weight changes of wintering *O. californica* bees from nicked cocoons were no more than those from intact cocoons across the range of provided humidities (paired *t*, *p =* 0.429). For wintering *M. rotundata* prepupae, wet weight changes at the more extreme humidities had the opposite expectation, with bees from nicked cocoons losing less water weight over Drierite and gaining more water weight at 75 and 88% humidity than those from intact cocoons.

Cocoons alone did exchange small amounts of moisture with the airspace. Cocoons that were first equilibrated at 35% humidity later lost 5.1% of their weight after a week at 1% humidity or gained 8.3% in weight at 88% humidity. However, individual cocoons only weigh about 0.01% of the adult bee (0.0125 mgs for *O. californica*). Consequently, cocoons contributed little to the measured weight change for the bee within its cocoon.

Most bees survived in every treatment to emerge as active adults. Among the treated *M. rotundata,* 93 of 96 individuals survived (3% mortality). All 32 bees subjected to the two extreme humidities survived, even including those bees from cut cocoons. More *O. californica* died, but their mortality was spread uniformly across the humidity treatments (3–5 dead out of 16 bees per each humidity treatment). The same proportions died whether or not their cocoons were intact (18%) or nicked (16%). Unexpectedly, nearly all of the dead *O. californica* bees were female (17 of 18 bees from 96 intact cocoons). In contrast, all but one male survived to emerge. Moreover, the females that died had initially been heavier than those that survived (195 vs. 149 mgs, Mann–Whitney U-test = 264, *p* < 0.004).

Deadwood tunnels in seemingly dry tree boles were quite humid in winter, averaging 75 ± 4.7% relative humidity (*n* = 10) at 12–23 °C. Water content of wood shavings collected during drilling averaged 18 ± 5.6%.

## 4. Discussion

Over the winter, storage humidity influenced the live weights of diapausing cocooned bees, both prepupae of *M. rotundata* (Figure 1) and adult *O. californica*. Water constituted a somewhat lesser fraction of their weights (Table 1) than the 65–75% typical of other diapausing insects [8]. Live weights changed the most during the first 6-week period. By 10 January, dormant *M. rotundata* had lost 2.05 ± 0.36% of their initial wet weight at 0% humidity, whereas at 88% humidity, they gained 5.4 ± 3.6% in wet weight (Figure 2). Wintering *O. californica* showed a comparable wet weight response to the range of provided humidities (Figure 3). This water weight response to aridity or high humidity early in winter diapause suggests that the bee within its cocoon passively equilibrated with moisture of the surrounding atmosphere. Active uptake would have led to some optimal body moisture content across humidity regimes. Inexplicably, only the tub with the K_2_CO_3_ salt solution had a measured relative humidity (55%) that did not match expectation (47%); it was also the only salt solution associated with anomalously large weight gains for both bee species (Figure 1, Figure 2 and Figure 3). Most likely, the solution was not fully saturated, yielding both the higher humidity and therefore greater weight gains of wintering bees.

Weight loss of wintering solitary bees is rarely measured. For *O. lignaria* held at an unknown humidity, prewintering bees lost ~20 mg (of a 165 mg bee), which was double their subsequent gradual weight loss over the winter (~5% of body weight) [9]. A comparable gradual weight decline over winter was reported in an earlier study [10]. That weight loss was attributed to fat body depletion, which was observed using X-radiograms and related to the bees’ respiration rates. Conversely, wintering prepupae of *M. rotundata* reportedly did not change weight over the winter [10], consistent with a wintering humidity of 35% in the current study (Figure 2). This contrast between the two species could be explained by winter storage humidity. If these bees were wintered at the relative humidity wherein bees’ body water content remained constant (Figure 1), then weight loss can be entirely attributed to fat body depletion (excepting generation of metabolic water). However, at higher or lower humidities, weight changes of diapausing bees will be functions of both fat body depletion and water loss or uptake from their storage atmosphere (Figure 2 and Figure 3). In natural deadwood nesting cavities in the arid western US, wood-nesting bees will gain a modest amount of water weight from the tunnel atmosphere (which averaged 75% in this study) (Table 1).

Cutting a small hole in some cocoons did not enhance weight change of the overwintering bees within, as might be expected if an intact cocoon served as an impervious barrier to water vapor exchange. Nonetheless, most of the cocoon surface does bar gas exchange, as shown using a novel apparatus that precisely measured cocoon gas exchange for one species each of *Osmia* and *Stelis* [11]. However, cocoons of many megachilid bees include a central anterior nipple-like process that holds an internal woven silk screen [11]. By comparing anterior and posterior ends for gas leak rates, that study showed that a cocoon’s nipple freely exchanged the tracer gas helium [11]. Air within the cocoon should freely exchange water vapor with the surrounding atmosphere through the cocoon nipple, explaining why weights of wintering bees in nicked versus intact cocoons were comparable for a given humidity.

Our drill hole airspaces in deadwood suitable for nesting were surprisingly humid in winter, averaging 75% RH. The experimental humidity over the saturated NaCl solution was most comparable. At that humidity, wintering bees only gained a little water weight (1.24% for *O. californica*; 2.2% for *M. rotundata*).

Bee mortality during the controlled wintering experiments was generally minor and independent of wintering humidity. The winter survival of *O. californica* was comparable to that reported for *O. lignaria* [5], including the nearly complete survival of males [12]. The greater winter mortality of female *O. californica—*mostly larger individuals—was unexpected and contradicts findings of studies with wintering *O. lignaria* [13]. Greater female wintering mortality could merely reflect the condition of our population prior to experiment. Nearly all *M. rotundata* survived at all humidities, even at the 0% humidity level maintained over a desiccant. Wintering resistance to desiccation by both *Megachile* and *Osmia* simplifies cold storage for managed cavity-nesting solitary bees. An easily maintained middling humidity will not detract from overwintering survival, although at the highest humidities, cocoon molding can become problematic.

The external source of water gain or loss of dormant bees must be respiratory or possibly trans-cuticular, as wintering bees neither feed nor defecate. Bodily water can also be generated internally as a metabolic byproduct. Prewintering insects, including bees [14], accumulate fat stores as triacylglycerides [15]. Completely metabolizing a gram of triacylglycerides yields about 1.1 g of metabolic water [16]. Thus, the relative humidity of the brood cell atmosphere and surrounding substrate can alter the wintering body water balance of *Osmia* and *Megachile*, and probably all other solitary bees of the temperate zones. Metabolic water then adds an unknown quantity. When the adult bee eventually emerges from its natal brood cell the following spring or summer, waste-laden water is ultimately excreted as liquid meconium. 

## Figures and Tables

**Table 1:** Water contents of immature diapausing bees in early winter.

Species	Life Stage	Sex	Fresh Wgt (mgs)	Water Wgt
*M. rotundata*	prepupa	mixed	30.1 ± 5	0.47
*O. californica*	adult	male	55.9 ± 7	0.55
*O. californica*	adult	female	117.7 ± 16	0.58

## Data Availability

The raw data supporting the conclusions of this article will be made available by the author on request.
